# Supplementary material for: Asynchronous magnetic resonance elastography: Shear wave speed reconstruction using noise correlation of incoherent waves
Source: Magn Reson Med. 2022 Oct 27;89(3):990–1001. doi: 10.1002/mrm.29502 (PMC9792433; doi:10.1002/mrm.29502)
Supplement: Supplementary file 1 — DATA S1 MATLAB code used to generate the numerical simulations of Figure 1 [file MRM-89-990-s002.zip › k-Wave/helpfiles/makeCircle.html]

makeCircle :: Functions (k-Wave)


# makeCircle

Create a binary map of a circle within a 2D grid.

## Syntax

```
circle = makeCircle(Nx, Ny, cx, cy, radius)
circle = makeCircle(Nx, Ny, cx, cy, radius, arc_angle)
circle = makeCircle(Nx, Ny, cx, cy, radius, arc_angle, plot_circle)
```

## Description

`makeCircle` creates a binary map of a circle or arc (using the midpoint circle algorithm) within a two-dimensional grid (the circle position is denoted by 1's in the matrix with 0's elsewhere). A single grid point is taken as the circle centre thus the total diameter will always be an odd number of grid points. The plot that is displayed after calling `makeCircle(128, 128, 64, 64, 30, (215/180)*pi, true);` is given below.

## Inputs

|  |  |
| --- | --- |
| `Nx, Ny` | size of the 2D grid [grid points] |
| `cx, cy` | centre of the circle [grid points], if set to 0, the centre of the grid is used |
| `radius` | circle radius [grid points] |

## Optional Inputs

|  |  |
| --- | --- |
| `arc_angle` | arc angle for incomplete circle [radians] (default = `2*pi`) |
| `plot_circle` | Boolean controlling whether the circle is plotted using `imagesc` (default = false) |

## Outputs

|  |  |
| --- | --- |
| `circle` | 2D binary map of a circle |

## Examples

- Using A Binary Sensor Mask
- Simulating Transducer Field Patterns
- Focussed Detector in 2D
- Focussed 2D Array with Directional Elements
- 2D Time Reversal For A Circular Sensor
- Image Reconstruction With Bandlimited Sensors
- Image Reconstruction With Compensation For Acoustic Absorption

## See Also

`makeCartCircle`, `makeDisc`
